# Road Traffic Injury Prevention Initiatives: A Systematic Review and Metasummary of Effectiveness in Low and Middle Income Countries

**DOI:** 10.1371/journal.pone.0144971

**Published:** 2016-01-06

**Authors:** Catherine Staton, Joao Vissoci, Enying Gong, Nicole Toomey, Rebeccah Wafula, Jihad Abdelgadir, Yi Zhou, Chen Liu, Fengdi Pei, Brittany Zick, Camille D. Ratliff, Claire Rotich, Nicole Jadue, Luciano de Andrade, Megan von Isenburg, Michael Hocker

**Affiliations:** 1 Emergency Medicine, Duke University Medical Center, Durham, North Carolina, United States of America; 2 Duke Global Health Institute, Duke University, Durham, North Carolina, United States of America; 3 Department of Nursing, State University of the West of Parana, Foz do Iguaçu, Parana, Brazil; University of New South Wales, AUSTRALIA

## Abstract

**Background:**

Road traffic injuries (RTIs) are a growing but neglected global health crisis, requiring effective prevention to promote sustainable safety. Low- and middle-income countries (LMICs) share a disproportionately high burden with 90% of the world’s road traffic deaths, and where RTIs are escalating due to rapid urbanization and motorization. Although several studies have assessed the effectiveness of a specific intervention, no systematic reviews have been conducted summarizing the effectiveness of RTI prevention initiatives specifically performed in LMIC settings; this study will help fill this gap.

**Methods:**

In accordance with PRISMA guidelines we searched the electronic databases MEDLINE, EMBASE, Scopus, Web of Science, TRID, Lilacs, Scielo and Global Health. Articles were eligible if they considered RTI prevention in LMICs by evaluating a prevention-related intervention with outcome measures of crash, RTI, or death. In addition, a reference and citation analysis was conducted as well as a data quality assessment. A qualitative metasummary approach was used for data analysis and effect sizes were calculated to quantify the magnitude of emerging themes.

**Results:**

Of the 8560 articles from the literature search, 18 articles from 11 LMICs fit the eligibility and inclusion criteria. Of these studies, four were from Sub-Saharan Africa, ten from Latin America and the Caribbean, one from the Middle East, and three from Asia. Half of the studies focused specifically on legislation, while the others focused on speed control measures, educational interventions, enforcement, road improvement, community programs, or a multifaceted intervention.

**Conclusion:**

Legislation was the most common intervention evaluated with the best outcomes when combined with strong enforcement initiatives or as part of a multifaceted approach. Because speed control is crucial to crash and injury prevention, road improvement interventions in LMIC settings should carefully consider how the impact of improvements will affect speed and traffic flow. Further road traffic injury prevention interventions should be performed in LMICs with patient-centered outcomes in order to guide injury prevention in these complex settings.

## Introduction

Road traffic injuries (RTIs) are a growing but neglected global health crisis, requiring effective prevention to promote sustainable safety. RTIs are the eighth leading cause of death, accounting for 75.5 million disability-adjusted life years (DALYs) globally in 2010 [1]. Every year, about 1.2 million people lose their lives on the roads and another 20–50 million people sustain non-fatal injuries due to road traffic crashes [2]. A disproportionately high burden of road traffic deaths and injuries occur in low and middle income countries (LMICs) (90%) and the burden is escalating due to rapid urbanization and motorization [2]. If appropriate actions are not taken, road traffic injuries are estimated to become the third leading cause of death and injury by 2020 [3].

Road traffic injuries are predictable and preventable. Despite the growing burden of RTIs globally, multiple intervention strategies and projects have contributed to a significant reduction of the burden of road traffic injuries in many high-income countries [4]. Empirical evidence for effective interventions is extensive, including enforcement of legislation on speed control and alcohol consumption, promotion of seatbelt and helmet utilization, and safer design and use of roads and vehicles [5]. A Global Plan for the Decade of Action for Road Safety 2011–2020 has been developed to guide efforts at national and local levels to reduce the forecasted level of road traffic fatalities around the world. The aim is to strengthen institutional capacity on road safety management and improve the health system for post-crash response [6]. Unfortunately, most of the data to date has been conducted in high-income settings and has focused on vehicle occupants rather than the highly vulnerable road users in LMICs [7].

Although several studies have assessed the effectiveness of a specific intervention through a systematic review approach [8], none have been conducted to provide policy makers and researchers with a summary of evidence on the comparative effectiveness of road traffic injury prevention interventions specifically performed in LMIC settings. While LMICs should avoid repeating high income country (HIC) studies showing the effectiveness of safety equipment (i.e. helmet use reduces deaths), it is imperative in resource-constrained settings to conduct pragmatic trials and comparative effectiveness studies to show the impact and cost of these safety measures in order to create strategic priorities. Therefore, the aim of this study is to identify road traffic injury prevention initiatives tested in LMICs in the literature and perform a metasummary to determine their effectiveness at reducing crashes, injuries or fatalities.

## Methods

### Protocol and Registration

This systematic review is reported in accordance with the Preferred Reporting Items for Systematic Review and Meta-Analyses (PRISMA) Statement [9], and is registered in the PROSPERO database (International Prospective Register of Systematic Reviews) under the number CRD42015019794.

### Eligibility Criteria

Our primary criteria for article consideration was evaluation of a road traffic injury prevention initiative conducted in a LMIC according to the World Health Organization criteria. To be included, articles had to be related to road traffic injury, evaluate a prevention-related intervention, evaluate an outcome (crash, injury, or death), and be peer-reviewed and published in English, Spanish, German, French, Swahili, Turkish, Arabic, or Chinese. Articles were excluded if they were abstracts, literature or systematic reviews, meta-analyses, or commentaries.

### Information Sources

We searched the electronic databases MEDLINE, EMBASE, Scopus, Web of Science, TRID, Lilacs, Scielo, and Global Health for articles that evaluated a road traffic injury intervention in a low- or middle-income country. Articles were chosen without any limitation on the language of the article. Additionally, we manually searched the references of the included articles and also performed a citation analysis of the included studies using Web of Science and Google Scholar for any citation appearing to meet the inclusion criteria based on the title and abstract.

### Search

The initial search comprised of the MeSH terms ‘Traffic Accident’ or ‘Injuries’ or ‘Low or Middle Income Countries.’ Appendix 1 demonstrates the search strategy used in PubMed, Embase, and Web of Science databases.

### Study Selection

We retrieved a total of 8560 articles which met the inclusion criteria. Five pairs of reviewers independently reviewed the titles and abstracts. Abstracts that did not provide enough information regarding the eligibility criteria were retrieved for full-text evaluation. Reviewers independently evaluated full-text articles and determined study eligibility. Disagreements were solved by consensus and if disagreement persisted, a third reviewer’s opinion was sought.

### Quality of Studies

Since our systematic review included studies of different designs (randomized control trials, non-randomized intervention, longitudinal with stepped wedge design, interrupted time-series, or secondary data/cross-sectional with before and after comparison) we opted to perform a data quality assessment according to study design using different approaches: (a) STROBE indicators for reporting observational studies; (b) two scales for non-randomized studies: the ACROBAT-NRS [10] and NOS [11]; (c) Cochrane's GRADE [12] mechanism for randomized studies; and (d) EPOC suggested risk of bias indicators for interrupted time series studies (EPOC) [13]. We assigned risk of bias (low, moderate and high risk) as suggested by the Cochrane Handbook [14] by study design. Studies were classified as high, moderate, and low risk of bias as such: (a) high risk when more than one indicator measure of bias was present across scales; (b) moderate risk when there is one indicator for bias across scales; (c) low risk when all indicators measure low or absence of bias. No studies were excluded from extraction based on risk of bias; however, studies’ contributions to the review results were analyzed in context of their risk of bias level.

### Data Extraction

Five pairs of reviewers independently conducted the data extraction and any disagreements that arose were resolved by a third reviewer. General characteristics of the studies, such as year of publication, location of study, and number of injuries or fatalities, were collected. In addition, information on health-related outcome measures, prevention type, prevention impact, or effectiveness measured as an effect size of outcome measures was extracted. The main outcome measures were head injury, overall injury, deaths, crashes, and helmet use.

### Data Analysis

Initial evaluation of the papers indicated that a meta-analytical approach would not be possible because of a high methodological variability (e.g. outcome measures, study designs, and sample characteristics). Therefore, we opted for a qualitative metasummary which aggregated qualitative findings from qualitative and quantitative studies [15] by grouping relevant findings from included studies into categories that represent the study's objectives, in our case, effectiveness of RTI prevention. The process involved in summarizing the main results of each included paper and performing a thematic analysis. Emerging themes,specifically types of interventions and main outcomes, were evaluated for each paper.

Effect sizes (ESs) are calculated to imply magnitude of emerging themes. ES value is calculated by dividing the number of studies containing a specific theme (excluding any duplicated findings) by the total number of included studies, resulting in a proportion expressed as percentage. Intensity of ES (IS) is then calculated to indicate which themes contributed more to answering the question (was more frequent among included papers) by dividing the number of findings contained in the study by the total number of findings across all studies, and which papers are "stronger" or "weaker" based on their contribution to answering the research question by dividing the number of findings with ESs >25% contained in that study by the number of findings with ESs >25% across all studies. This information is useful to improve interpretation based on a qualitative metasummary approach. It also helps to determine whether any findings were derived largely from “weaker” studies, which reports contributed most of the findings with the largest frequency effect sizes across reports, and which reports contained findings no other reports contained [16].

### Reproducibility

This paper followed the framework for reproducible research reports [17]. Dataset (in.csv format) and analysis codes are shared through our github repository (joaovissoci/prevention_sr). The codes are linked to the data set and functional. All this material and other information can also be found gathered in our project's website (https://sites.google.com/site/dghiroadtrafficinjurypreventio/). All documents are licensed with Creative Commons Attribution, Non-commercial 3.0 License.

## Results

### Study Selection and Description

**Fig 1:**
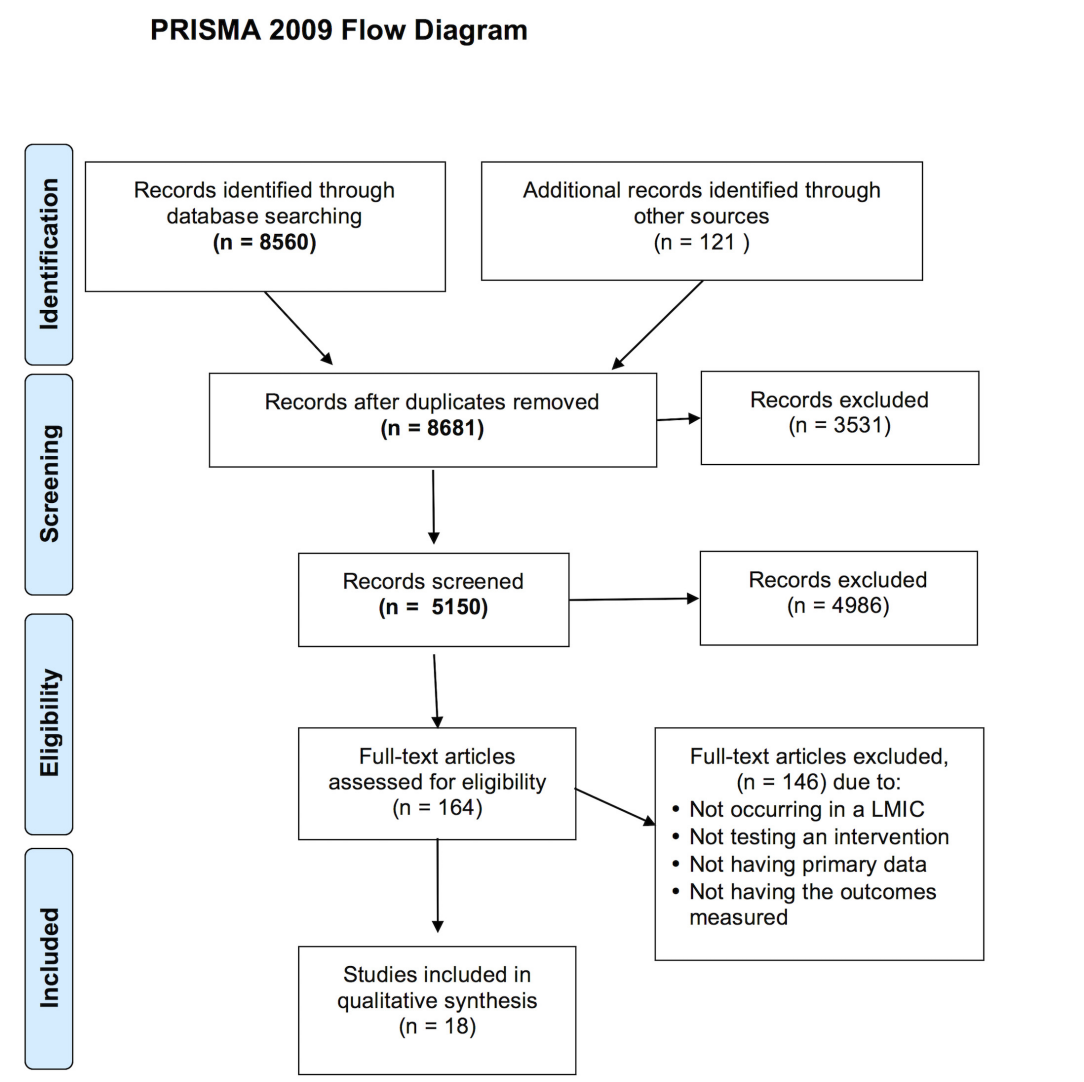
Study flow diagram.

Through a database search, 8560 articles were initially identified which yielded 18 final articles that fit the inclusion/exclusion criteria “Fig 1”.

**Fig 2:**
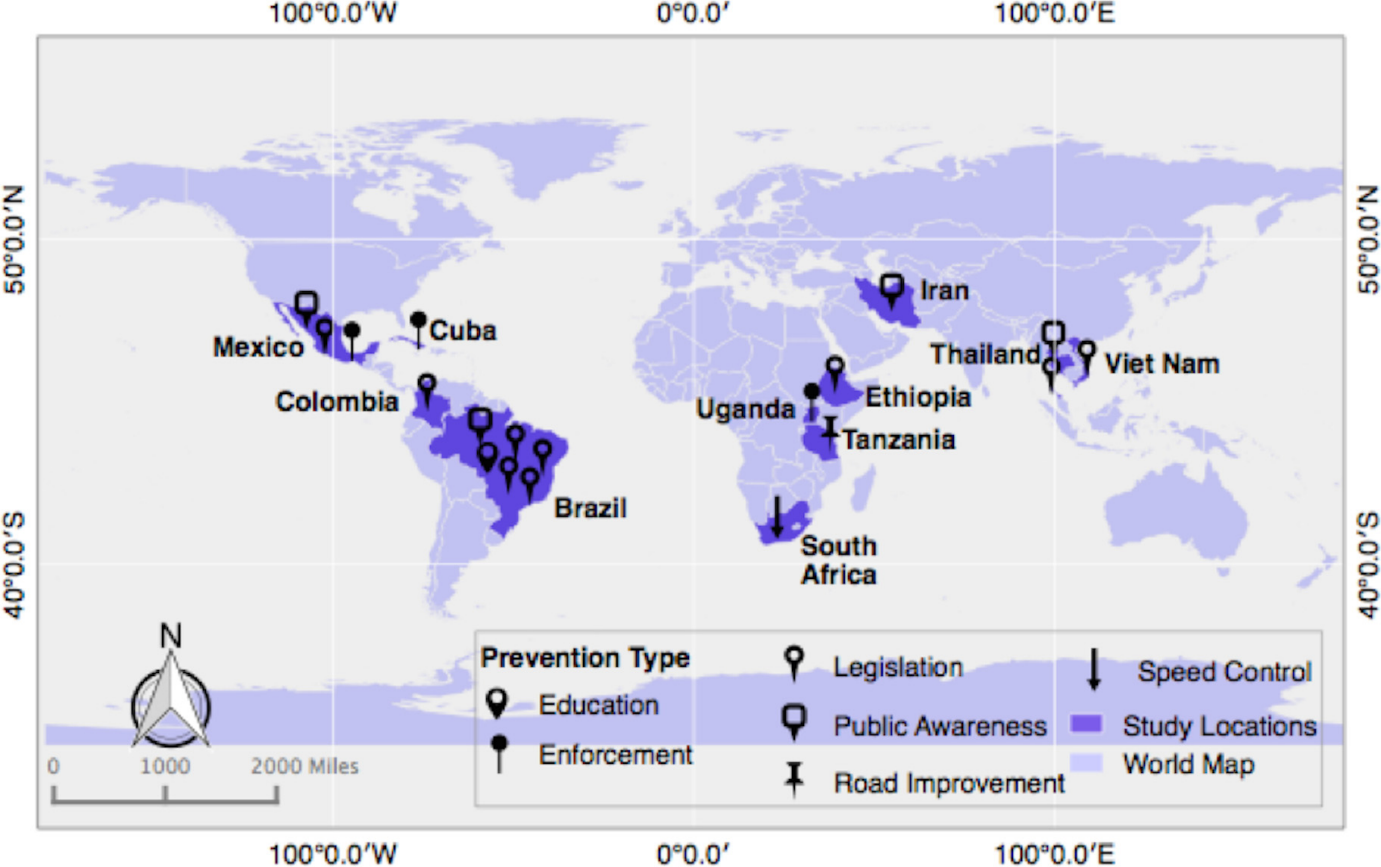
Map of the location and type of intervention evaluations included.

Of these 18 included studies, we extracted 19 types of intervention. Four studies were from Sub-Saharan Africa, 10 from Latin America and the Caribbean (a study from Mexico had two different types of interventions), one from the Middle East, and three from Asia “Fig 2”. Half of the studies focused specifically on legislation (9), while the others focused on speed control measures (1), public awareness/education (4), enforcement (2), road improvement (1), and a multifaceted intervention (with legislation and education interventions) [18]. The characteristics of each manuscript are described further in Table 1 and the intervention types and summary of outcomes are included in Table 2.

**Table 1:** Road Traffic (RT) Injury Prevention Study Characteristics.

Authors	Geographic Region	Prevention Type	Study Design	Targeted Population	Risk of Bias	Outcome Measures
Abegaz et al., 2014 [19]	Ethiopia	Legislation	Interrupted time series	All road users	Low	RTC per 10,000 vehicles,RT deaths per 10,000 vehicles
Andreuccetti et al., 2011 [20]	Brazil	Legislation	Interrupted time series	Vehicle drivers	Low	RT injuries,RT deaths
Bacchieri et al., 2010 [21]	Brazil	Education	Non-randomized intervention—Longitudinal with steeped wedge design	Cyclists	Low	Cyclist crashes,Cyclist 'near miss'
Bishai et al., 2008 [22]	Uganda	Enforcement	Interrupted time series/Cost-Effectiveness	All road users	Low	RT deathsRT crashes
Chandran et al., 2014 [18]	Mexico	Multifaceted (legislation and education)	Interrupted time series	All road users	Low	RT crashes,RT injuries,RT deaths
de Andrade et al., 2008 [23]	Brazil	Legislation	Interrupted time series	All road users	Low	RT deaths
Espitia-Hardeman et al., 2008 [24]	Colombia	Legislation	Interrupted time series	Motorcyclists	Low	RT deaths
Farage et al., 2002 [25]	Brazil	Legislation	Cross-sectional	All road users	Moderate	TBI cases,RT crashes,RT deaths
Gómez-García et al., 2014 [26]	Mexico	Legislation	Interrupted time series	All road users	Low	Alcohol RT crashes, Alcohol-related RT injuries
Guanche Garcell et al., 2008 [27]	Cuba	Enforcement	Cross-sectional/Time-series	Vehicle drivers	High	RT crashes,RT deaths,RT injuries
Ichikawa et al., 2003 [28]	Thailand	Legislation	Interrupted time series	Motorcyclists	Low	Motorcycle crashes
Nadesan-Reddy & Knight, 2013 [29]	South Africa	Speed Control	Interrupted time series	All road users	High	Pedestrian/Vehicle RT crashes
Passmore et al., 2010 [30]	Viet Nam	Legislation	Interrupted time series	All road users	Moderate	Risk for head injuries among RT injuries patients, RT deaths
Poli de Figueiredo et al., 2001 [31]	Brazil	Legislation	Interrupted time series	All road users	Moderate	RT crashes,RT deaths
Rahimi-Movaghar, 2010 [32]	Iran	Community	Cross-sectional/Community comparison	All road users	Moderate	RT injuries,RT deaths
Salvarani et al., 2008 [33]	Brazil	Education	Interrupted time-series	Vehicle drivers	Moderate	RT injuries,RT trauma severity,RT deaths
Swaddiwudhipong et al., 1998 [34]	Thailand	Education	Community RCT	Motorcyclists	Low	RT injuries
Zimmerman et al., 2015 [35]	Tanzania	Road Improvement	Non-randomized intervention Longitudinal with steeped wedge design	All road users	Low	RT injury rate

**Table 2:** Intervention description and summary of outcomes.

Intervention Type	Intervention Description	Summary of Outcomes
**Legislation** [19] [20] [28] [23] [31] [26] [30] [24] [25]	Improved road safety policy with laws and higher penalties	Overall RTI reduction range from 1.8% to 33.5% in all road users and 10.5% in motorcycle drivers.
	Blood alcohol concentration (BAC) level reduction	
		Specifically, head injury reduction of 16% to 33% in motorcyclists.
	Legislation on helmet, seat belt, and cell phone use	
		Hospital length of stay reduction of 14.7%. RT-related ER admissions reduced 17.7% to 33% within road users. Alcohol-related death reduction of 5.7%.
	Increase of penalty and application of a scoring system to license withdrawal	
		Crash reduction range from 10.5% to 21.3%.
	Death reduction range from 7.2% to 33.2% in all road users. Motorcycle drivers had reports of reduction in 5.7 deaths/month and 7.1% to 16.4% in deaths. Some reports showed non-significant reduction of death in motorcyclists.	
		Time trend showing change right after law implementation and monthly rate decrease of 5.1 crashes and 1.96 deaths, with reports of non-significant time trend change in mortality.
**Enforcement** [22] [18] [27]	Increase of police enforcement	Death reduction of 17% or 118 lives saved, reduction in crashes and fatal crashes.
	Training on technology for enforcement	
		No significant reduction to death and injuries, but decrease of 58% in crash frequency.
	Setting target enforcement practices	
**Public Awareness/ Education** [21] [32] [34] [18] [33]	Application of the WHO Safe Community model	No significant difference in injury trends or in crashes or alcohol use in motorcycle drivers.
	Education for motorcyclists/ bicyclists	Reduction of 21% in RT deaths in all road users. Increase of mild and decrease in moderate (16%) and severe trauma (51%).
	Social marketing with mass media and road signs	Injury deaths decrease (23.3%) compared to control communities among all road users.
	Education and training using health centers and schools/universities	Reduction of 18.3% in near miss accidents with cyclists and approximately 50% reduction in incidence.
**Speed Control** [36] [29]	Speed control evaluated by interventions of harder legislation for speed control and insertion of rumble strips (speed bumps)	No significant crash reduction regarding speed legislation.
		Rumble strips decreased RT fatalities from 55% to 68%, crashes by 33%, and pedestrian-vehicle collisions by 23%.
**Road Improvement** [35]	Comparison of a repaved with an unpaved road	RTI significantly higher in intervention compared to the control.

#### Quality of Studies

Our quality of studies evaluation indicated that seven studies had at least one bias indicator. Specifically, the main risk of bias common among the studies was a lack of appropriate data analysis methods for studies with a time-series design. Several studies conducted an observational study with an interrupted time-series design but did not use a time-series analytical approach (e.g. ARIMA models) [27] [29] [30] pooling the pre- and post-intervention data, or they did not apply an inferential statistical approach [31] [33]. Other sources of bias found were not clearly indicating data sources [27], and sample size adequacy and/or characteristics [29] [32]. In relation to data quality, since most included studies dealt with secondary data analysis, one common issue was failure to address underreporting or missing data practices, which is a major concern with registry data in LMICs. However, 11 studies reported good quality in relation to the risk of bias. Based on these results and the heterogeneity of study characteristics, our option to report descriptively is justified and the risk of observed bias has only a small impact on our results. Most studies report a reduction in the prevalence of deaths, crashes, or RTIs. Few studies report the prevalence of hospital admissions or alcohol.

#### Synthesis of RTI Prevention Studies

**Fig 3:**
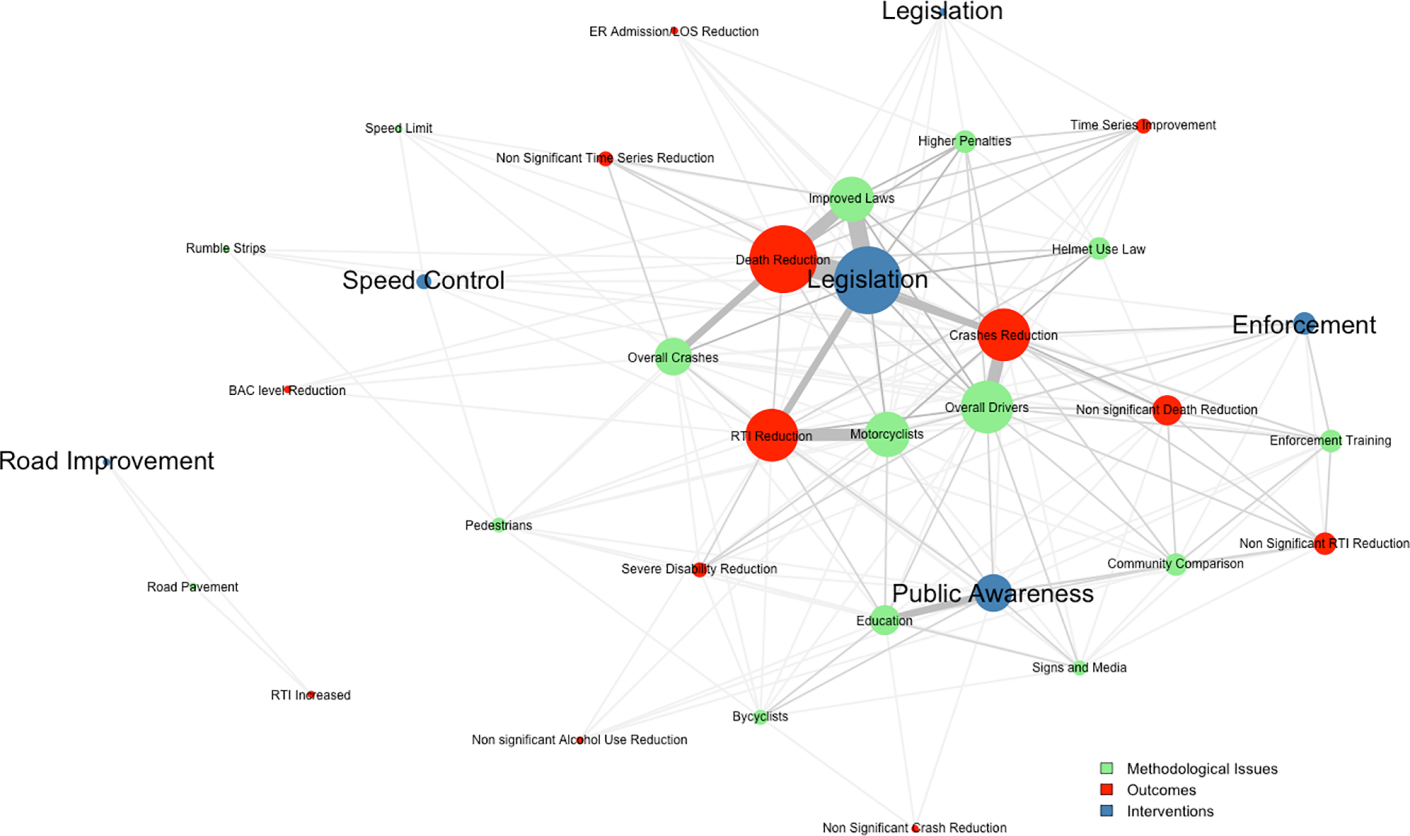
Synthesis of Road Traffic Interventions and Outcomes.

Metasummary results are presented in “Fig 3”, showing a larger ES for legislation followed by public awareness and enforcement. Death reduction was the most intense emerging outcome theme related to RTI prevention initiatives, followed by crash reduction and RTI reduction. Through the network display, it is clear that most significant outcomes are directly linked to legislation, while the other intervention methods are more inconsistent related to outcome. Most research focused on the type of crash, number of road crashes in a defined time period, and reported fatalities or injuries. Surprisingly, road improvements may increase road traffic injuries Each emerging prevention theme is discussed individually below.

#### Legislation

Nine studies evaluated the impact of legislation intervention on RTI outcomes. Early data from Brazil in 1995 [25] investigated official registries to evaluate the effect of legislation on seat belt usage and speed limit legislation, and found an increase in traumatic brain injuries and RTC but a decrease in deaths, although no statistical measures were applied. A second study from Brazil [31] analyzed data from emergency care visits for RTIs before and after the introduction and enforcement of the 1998 Brazilian Traffic Code. While only an association, the authors found a 24.7% drop in immediate road traffic fatalities and a 21.3% reduction in crash incidence.

Another Brazilian study evaluating the Brazilian Traffic Code [23] found that while traffic crash mortality rates initially decreased, these rates began to rise again after legislation had been in place for over a year. Similar legislation was passed in Ethiopia [19], and the combination of improving current road legislation and adding new road safety laws (mandatory motorcycle helmet usage, banning cellular phone usage when driving, and seat belt laws) resulted in a reduction of deaths, overall injury, and crashes.

Legislative measures implementing a single road safety measure may also be effective. One study in Brazil [20] suggested that legislation decreasing the legal blood alcohol content (BAC) level from 0.06 g/L to 0.02 g/L was associated with a significant (p<0.05) reduction in both traffic fatalities (7.2% to 16%) and injuries (1.8% to 2.3%). As expected, areas with higher levels of police enforcement demonstrated higher levels of effective legislation. Similarly, in 2010, Mexico also implemented legislation to reduce the legal BAC to 0.05 g/L, with increasing penalties with increasing BAC [26] [24]. This legislation was associated with a reduction of 5.7% in alcohol-related deaths, with an atypical 0% deaths in December 2010 and July 2011. A significant reduction in crashes was also observed.

Results from motorcycle drivers specifically showed that in Colombia (1993–2001), a sequence of legislation about safe habits showed an impact in deaths of 12.3% to 16.4% in the following year [24]. However, this effect was decreased after initial impact and due to a discontinued enforcement. Ultimately, as a conjoint analysis, deaths decreased in a rate of 5.7 deaths/month. In 1996, Thailand implemented a mandatory helmet law for motorcyclists. A study to evaluate any effect of this legislative change compared motorcyclist fatalities and injuries at a regional hospital from January 1994 to December 1997 and found that while fatalities did not decrease, there was a 33.5% reduction in the number of motorcycle-related injuries, and injuries seen were less severe [28]. Not specifying the driver population, a report from Viet Nam also tested the impact of a helmet use law in 2007 [30] [24]. The result showed a difference from before and after crash prevalence of 10.5%, while risk for deaths decreased 18%. They also observed a decrease of 16% in the risk for RTI head injury.

#### Enforcement

Two studies focused in particular on the enforcement of existing road traffic safety measures. One study in Uganda increased police presence on four major roads in the capital city of Kampala; results found a point reduction of 17% in the number of road traffic deaths on these roads [22]. Another study in Mexico examined the effect on increasing drunk-driving law enforcement while also increasing seat-belt awareness campaigns in two major cities; this involved a focus on enforcement during the first year and the addition of the awareness campaign in the second year. Results revealed a decrease in road traffic crashes after the first year, but no significant reduction in deaths or injuries was seen in either year [18].

#### Public Awareness/Education

Five studies examined the effectiveness of public awareness and educational campaigns. In Mexico, an awareness campaign targeting seat belt and child restraint use was added in the second year of a project after a one-year law enforcement campaign. Similar to the evaluation results after the first year of the study, the public awareness campaign had a reduction in RTC but not in fatalities or injuries. Another study, in Brazil, performed a multifaceted public awareness/education intervention campaign [33] focusing on educational training in health centers and schools/universities, and public awareness campaign with media distribution of videos, souvenirs, and pamphlets. Results showed small increases in outcomes such as crashes and injuries, but also found a 26% decrease in deaths as well asmoderate and severe trauma. Also, the same study reported a 25.6% decrease in ICU admissions due to RTI.

Two other educational interventions evaluated in Brazil and Thailand consisted of pre-post surveys with control groups and an educational intervention which focused on safety and injury rates for bicyclists and motorcyclists, respectively [34] [21]. Results support that education interventions reduced overall injuries (10.1% fewer injuries), crashes (12.0% fewer crashes), and increased helmet use (25.5% more helmet use). No impact was observed on deaths. In the Brazilian bicyclist study, however, low attendance was also an issue, with only 45% of cyclists attending the educational program.

Finally, one article evaluated the introduction of the World Health Organization Safe Community (SC) model in an Iranian county [37]. Evaluation of this intervention compared the injuries reported at the emergency department of the intervention county and 44 other counties in Iran. When compared to the other counties, there was a significant decrease in injury rate (23.3%) for three years after implementing the SC model [32].

#### Speed Control

Traffic calming bumps, or speed bumps, are also another method of speed control. In a South African study, an interrupted time-series study examined the effect of speed bumps in two different residential locations. The addition of speed bumps reduced the number of serious pedestrian-vehicle collisions by 22% and 23% while fatal pedestrian-vehicle collisions decreased by 50% and 68% in the two different locations [29].

#### Road Improvement

One study examined improvement of road quality through the paving of a highway. This Tanzanian study compared reported injuries in two separate rural communities before and after one community paved the highway. The study found that after the road was paved, RTIs increased in that community; an increase was also seen in the control community, but it was not as significant [35].

## Discussion

This is the first systematic review on RTI prevention initiatives’ effectiveness in LMICs. This review found 18 manuscripts from 11 LMICs focusing on legislation, enforcement, public awareness/ education, speed control and road improvement. These studies found legislation-based interventions had the strongest evidence for road traffic crash, injury, and death reduction. This comprehensive review highlights the limited number and quality of manuscripts on this topic and calls for more evaluations of RTI prevention initiatives in LMICs on patient-centered outcomes in order to inform policy makers’ priorities.

Given the known high burden of RTI which rests especially upon LMICs [38], there is limited evidence for effective interventions in these settings to guide future implementation priorities. With 34 low income and 105 middle income economies, to have RTI prevention intervention evaluations conducted in only 11 countries highlights the limited research in this field [39]. There have been numerous calls for increased literature focusing on RTI, especially in the setting of the UN’s Decade of Action for Road Traffic Injury Prevention 2010–2020 [40]. Similarly, multiple journals have published calls for research [41] [42], yet there has been a relatively small increase in the literature found by this review at the middle of this decade of action. While there is an abundance of literature available in HIC about the effectiveness and cost-effectiveness of RTI injury prevention initiatives (like helmet use and legislation), the adaptation, implementation, and evaluation of these interventions to a LMIC setting is still missing. Given the complexities of LMIC settings, the effectiveness and cost of certain interventions in LMIC settings will allow for prioritization that takes into account the limited infrastructure, implementation challenges, and limited funding inherent in these settings.

The majority of studies found were time-series and, while often methodologically sound and of high quality, 7 of the 18 studies reported at least one indicator of bias, the most common being a lack of appropriate data analysis methods for the time-series design. Other sources of bias found included not clearly indicating data sources, sample size adequacy and/or characteristics, and failure to address underreporting or missing data practices, which are likely common occurrences in registry data of LMICs. Most studies relied on police, hospital records, and trauma registries, all of which reported a reduction in the prevalence of RTCs, injuries, or deaths. Performing a meta-analysis was extremely difficult given the multiple types of data sources, outcomes tested, and differing interventions. This also highlights the difficulty with comparing data from different locations. Similarly, the types of studies available show the challenges of performing randomized trials and patient outcome–centered trials in RTI prevention in LMICs. As we continue to promote RTI prevention through research, mixed-methods and advanced trials as well as standardized outcomes will be needed to further address challenges in behavior change. For example, a study evaluating the effectiveness of a road traffic awareness safety program in Ghana cited qualitative data on how people perceived the messaging, but failed to measure any comparable outcomes, such as traffic injury rates [43].

Our results suggest that all types of intervention, other than road improvement, had some data supporting an impact, albeit sometimes short-lived, on road traffic crashes, injuries, and fatalities. Legislative interventions did have the most number of supporting articles as well as the strongest evidence of reduction in crashes, injuries, and fatalities. In most of these settings, the amount of safety or road use legislation is limited, so the potential benefit of well-designed enforceable legislation is likely immense. A key message of our findings, and numerous other sources, was that enforcement of legislation had an impact on the intervention success and sustainability [44] [31] [45]. Concerningly, educational interventions, which are commonly adopted due to low cost and low intervention complexity, in our review had limited effect. These findings mirror findings from HIC which suggest that educational interventions have limited evidence of effectiveness at injury or fatality reduction [46] [47]. Attendance and behavior change from educational interventions are limited [48] [21] even in the setting of legislation. As such, interventions should be multifaceted, focusing on education as well as legislation and should be closely evaluated for the enforcement challenges and possibilities to increase their impact.

While the World Health Organization has suggested road safety audits, Zimmerman found that road improvements lead to increased RTI [38] [35]. Unfortunately, the impact of Zimmerman’s road improvement was possibly higher vehicular speeds, which have been cited as a major cause of RTI. While speed control with speed bumps has been found to be protective against injuries, there were only two studies on these environmental changes in a LMIC [36] [29]. Some studies attempting to improve the road environment by changing pedestrian behavior to utilize bridges or underpasses failed due to perceived risks and inconvenience and instead led pedestrians to increase their personal risk by creating their own path through traffic [49] [50] [51]. Further evaluations of road safety audits and implementation initiatives should be undertaken, especially in the complex road environments found in LMIC.

### Limitations

This study did have limitations which must be taken into account when evaluating our results. Given our inclusive methodology, we ended up with multiple types of study design and outcomes which made comparing the studies and performing a quantitative meta-analysis impossible. However, if we had narrowed the types of studies to include only one type of study, we would not have had enough studies to evaluate and conduct a meta-analysis given the paucity of literature with similar methodologies on this topic. In an effort to find evidence that showed a reduction in injuries/deaths rather than the adoption of a safety intervention only, we choose patient-centered outcomes like injury or mortality rates. While this information presents a stronger argument for prevention, it is also more difficult to capture in a LMIC setting with limited data gathering infrastructure; as such, these manuscripts are less prevalent. The limited number of articles identified could be indicative of the limitations in funding for implementation, evaluation, and publication about road traffic injury prevention initiatives. Therefore, numerous other successful RTI prevention initiatives may be available in the grey literature and not in the standard literature. We didn’t include the grey literature, as we wanted to highlight those that have undergone the academic rigor of peer reviewed publication.

## Conclusion

Our systematic review found a total of 18 articles that make up the evidence for road traffic injury prevention initiatives evaluated in LMIC settings. Legislation interventions were the most common and reduced road traffic crashes, injuries and deaths with the best results in the setting of good enforcement initiatives. As expected, with legislation as well as education and public awareness campaigns, a significant reduction in fatalities appeared immediately following enactment with non-significant decreases over time. While public awareness and speed control interventions alone appeared to have no significant effects on reducing road traffic injuries or fatalities, when combining them into a multifaceted approach, they were shown to be more effective at significantly reducing road traffic fatalities and injuries over time. Because speed control is crucial to crash and injury prevention, road improvement interventions should consider how the impact of improved roads will affect speeds and traffic flow. In LMICs where enforcement and resources are limited, rumble strips could be effective at reducing road traffic crashes and fatalities through speed control. Further road traffic injury prevention interventions should be performed in LMICs with patient-centered outcomes in order to guide interventions in these complex settings.

## Supporting Information

S1 Table Characteristics. (CSV)

S2 Table Intervention Based Summary. (CSV)

S3 Table Map. (CSV)

S4 Table PubMed Search. (DOCX)

S5 Table Embase Search. (DOCX)

S6 Table Scopus Search. (DOCX)

S7 Table PRISMA Checklist. (DOC)

## References

[1] Murray CJ, et al, Disability-adjusted life years (DALYs) for 291 diseases and injuries in 21 regions, 1990–2010: a systematic analysis for the Global Burden of Disease Study 2010. Lancet, 2012 380(9859): p. 2197–223. 10.1016/S0140-6736(12)61689-4 23245608

[2] Organization WH. WHO global status report on road safety 2013: supporting a decade of action 2013, World Health Organization.

[3] Murray CJ, Lopez AD. *The global burden of disease and injury series*, *volume 1: a comprehensive assessment of mortality and disability from diseases*, *injuries*, *and risk factors in 1990 and projected to 2020*. Cambridge. MA, 1996.

[4] Organization WH. World Report on Road Traffic Injury Prevention; 2004 2008, World Health Organization.: Geneva.

[5] Peden M, et al World report on road traffic injury prevention World Health Organization. in Hyder AA, Jarawan E, Mathers C. 2004 Citeseer.

[6] Organization WH. Saving millions of lives: decade of Action for Road Safety 2011–2020 2011, World Health Organization.

[7] Ameratunga S, Hijar M, Norton R. Road-traffic injuries: confronting disparities to address a global-health problem. The Lancet, 2006 367(9521): p. 1533–1540. 10.1016/S0140-6736(06)68654-6 16679167

[8] Porchia B.R., et al, Effectiveness of two interventions in preventing traffic accidents: a systematic review. Ann Ig, 2014 26(1): p. 63–75. 10.7416/ai.2014.1959 24452185

[9] Moher D., et al, Preferred reporting items for systematic reviews and meta-analyses: the PRISMA statement. PLoS Med, 2009 6(7): p. e1000097 10.1371/journal.pmed.1000097 19621072 PMC2707599

[10] Sterne, J., J. Higgins, and B. Reeves, *on behalf of the development group for ACROBAT-NRSI* *A cochrane risk of bias assessment tool: for non-randomized studies of interventions (ACROBAT-NRSI)*, *version 1**0* *0*, *24 September 2014*. 2014.

[11] Wells G., et al, The Newcastle-Ottawa Scale (NOS) for assessing the quality of nonrandomised studies in meta-analyses University of Ottawa 2001, Accessed 12/16/2009 at http://www.ohri.ca/programs/clinical_epidemiology/oxford.htm.

[12] Higgins J.P., et al, The Cochrane Collaboration’s tool for assessing risk of bias in randomised trials. Bmj, 2011 343. 10.1136/bmj.d5928 PMC3196245 22008217

[13] Services, N.K.C.f.t.H. *Effective Practice and Organisation of Care (EPOC)* EPO Resources for Review Authors 2015; Available: http://epoc.cochrane.org/epoc-specific-resources-review-authors.

[14] Higgins J., Green S. Cochrane Handbook for Systematic Reviews of Interventions Version 5.0 *0 [updated February 2008]* The Cochrane Collaboration, 2008 www.cochrane-handbook.org, 2013.

[15] Sandelowski M., Barroso J., and Voils C.I., Using qualitative metasummary to synthesize qualitative and quantitative descriptive findings. Res Nurs Health, 2007 30(1): p. 99–111. 17243111 10.1002/nur.20176 PMC2329806

[16] Sandelowski M. and Barroso J., Creating metasummaries of qualitative findings. Nurs Res, 2003 52(4): p. 226–33. 12867779 10.1097/00006199-200307000-00004

[17] Vissoci, J.R.N., et al., *A Framework for Reproducible*, *Interactive Research: Application to health and social sciences*. arXiv preprint arXiv:1304.5688, 2013.

[18] Chandran A., et al, Early impact of a national multi-faceted road safety intervention program in Mexico: results of a time-series analysis. PLoS One, 2014 9(1): p. e87482 10.1371/journal.pone.0087482 24498114 PMC3909119

[19] Abegaz T., et al, Effectiveness of an improved road safety policy in Ethiopia: an interrupted time series study. BMC Public Health, 2014 14: p. 539 10.1186/1471-2458-14-539 24886220 PMC4052816

[20] Andreuccetti G., et al, Reducing the legal blood alcohol concentration limit for driving in developing countries: a time for change? Results and implications derived from a time-series analysis (2001–10) conducted in Brazil. Addiction, 2011 106(12): p. 2124–31. 10.1111/j.1360-0443.2011.03521.x 21631625 PMC3184361

[21] Bacchieri G., et al, A community intervention to prevent traffic accidents among bicycle commuters. Rev Saude Publica, 2010 44(5): p. 867–75. 20877924 10.1590/s0034-89102010000500012

[22] Bishai D., et al, Cost-effectiveness of traffic enforcement: case study from Uganda. Inj Prev, 2008 14(4): p. 223–7. 10.1136/ip.2008.018341 18676779

[23] Maffei de Andrade S., et al, Road injury-related mortality in a medium-sized Brazilian city after some preventive interventions. Traffic Inj Prev, 2008 9(5): p. 450–5. 10.1080/15389580802272831 18836956

[24] Espitia-Hardeman V., et al, [Impact of interventions directed toward motorcyclist death prevention in Cali, Colombia: 1993–2001]. Salud Publica Mex, 2008 50 Suppl 1: p. S69–77. 18373012 10.1590/s0036-36342008000700011

[25] Farage L., et al, [Safety measures in traffic and hospital morbimortality in craniocerebral trauma in the Distrito Federal]. Rev Assoc Med Bras, 2002 48(2): p. 163–6. 12205535 10.1590/s0104-42302002000200037

[26] Gomez-Garcia L., Perez-Nunez R., and Hidalgo-Solorzano E., [Short-term impact of changes in drinking-and-driving legislation in Guadalajara and Zapopan, Jalisco, Mexico]. Cad Saude Publica, 2014 30(6): p. 1281–92. 25099051 10.1590/0102-311x00121813

[27] Guanche Garcell H., et al, [Impact of a drink-driving detection program to prevent traffic accidents (Villa Clara Province, Cuba)]. Gac Sanit, 2008 22(4): p. 344–7. 18755085 10.1157/13125356

[28] Ichikawa M., Chadbunchachai W., and Marui E., Effect of the helmet act for motorcyclists in Thailand. Accid Anal Prev, 2003 35(2): p. 183–9. 12504139 10.1016/s0001-4575(01)00102-6

[29] Nadesan-Reddy N. and Knight S., The effect of traffic calming on pedestrian injuries and motor vehicle collisions in two areas of the eThekwini Municipality: a before-and-after study. S Afr Med J, 2013 103(9): p. 621–5. 10.7196/samj.7024 24300678

[30] Passmore J., et al, Impact of mandatory motorcycle helmet wearing legislation on head injuries in Viet Nam: results of a preliminary analysis. Traffic Inj Prev, 2010 11(2): p. 202–6. 10.1080/15389580903497121 20373241

[31] Poli de Figueiredo L.F., et al, Increases in fines and driver licence withdrawal have effectively reduced immediate deaths from trauma on Brazilian roads: first-year report on the new traffic code. Injury, 2001 32(2): p. 91–4. 11223038 10.1016/s0020-1383(00)00172-8

[32] Rahimi-Movaghar V., Controlled evaluation of injury in an international Safe Community: Kashmar, Iran. Public Health, 2010 124(4): p. 190–7. 10.1016/j.puhe.2010.02.014 20417350

[33] Salvarani C.P., Colli B.O., and Carlotti C.G. Junior, Impact of a program for the prevention of traffic accidents in a Southern Brazilian city: a model for implementation in a developing country. Surg Neurol, 2009 72(1): p. 6–13; discussion 13–4. 10.1016/j.surneu.2007.10.008 18328548

[34] Swaddiwudhipong W., et al, Effect of motorcycle rider education on changes in risk behaviours and motorcycle-related injuries in rural Thailand. Trop Med Int Health, 1998 3(10): p. 767–70. 9809909 10.1046/j.1365-3156.1998.00301.x

[35] Zimmerman K., et al, Road traffic injury on rural roads in Tanzania: measuring the effectiveness of a road safety program. Traffic Inj Prev, 2015 16(5): p. 456–60. 10.1080/15389588.2014.973491 25356935

[36] Afukaar F.K., Speed control in developing countries: issues, challenges and opportunities in reducing road traffic injuries. Inj Control Saf Promot, 2003 10(1–2): p. 77–81. 12772489 10.1076/icsp.10.1.77.14113

[37] Spinks A., et al, The 'WHO Safe Communities' model for the prevention of injury in whole populations. Cochrane Database Syst Rev, 2005(2): p. CD004445 15846716 10.1002/14651858.CD004445.pub2

[38] Organization W.H., Global status report on road safety: time for action 2009: World Health Organization.

[39] Bank, T.W., *Country and Lending Groups*. 2015, The World Bank: data.worldbank.org.

[40] Harris M., Call for a Decade of Action for Road Safety 2010–2020: the first Global Ministerial Conference on road safety and beyond, in Road safety data: collection and analysis for target setting and monitoring performances and progress. 2009, International Transport Forum: Australia.

[41] Sharma B.R., Road traffic injuries: a major global public health crisis. Public Health, 2008 122(12): p. 1399–406. 10.1016/j.puhe.2008.06.009 18950819

[42] Hyder A.A. and Peden M., Inequality and road-traffic injuries: call for action. Lancet, 2003 362(9401): p. 2034–5. 14697797 10.1016/S0140-6736(03)15145-8

[43] Blantari J., et al, An evaluation of the effectiveness of televised road safety messages in Ghana. Int J Inj Contr Saf Promot, 2005 12(1): p. 23–9. 15814372 10.1080/17457300512331342199

[44] Ameratunga S., Hijar M., and Norton R., Road-traffic injuries: confronting disparities to address a global-health problem. Lancet, 2006 367(9521): p. 1533–1540. 16679167 10.1016/S0140-6736(06)68654-6

[45] WPoTRE, E.T.S.C. *Police enforcement strategies to reduce traffic casualties in Europe*. 1999.

[46] Davies G.R. and Roberts I., Is road safety being driven in the wrong direction? Int J Epidemiol, 2014 43(5): p. 1615–23. 24808047 10.1093/ije/dyu103

[47] Duperrex O., Roberts I., and Bunn F., Safety education of pedestrians for injury prevention. Cochrane Database Syst Rev, 2002(2): p. CD001531 12076415 10.1002/14651858.CD001531 PMC7025789

[48] Sumner S.A., et al, Effect of free distribution of safety equipment on usage among motorcycle-taxi drivers in Tanzania—A cluster randomised controlled trial. Injury, 2014 45(11): p. 1681–6. 10.1016/j.injury.2014.04.034 24861418 PMC4213314

[49] Hijar M., Vazquez-Vela E., and Arreola-Risa C., Pedestrian traffic injuries in Mexico: a country update. Inj Control Saf Promot, 2003 10(1–2): p. 37–43. 12772484 10.1076/icsp.10.1.37.14108

[50] Forjuoh S.N., Traffic-related injury prevention interventions for low-income countries. Inj Control Saf Promot, 2003 10(1–2): p. 109–18. 12772494 10.1076/icsp.10.1.109.14115

[51] Mutto M., Kobusingye O.C., and Lett R.R., The effect of an overpass on pedestrian injuries on a major highway in Kampala—Uganda. Afr Health Sci, 2002 2(3): p. 89–93. 12789091 PMC2141581

