# Correction: Road Traffic Injury Prevention Initiatives: A Systematic Review and Metasummary of Effectiveness in Low and Middle Income Countries

**DOI:** 10.1371/journal.pone.0150150

**Published:** 2016-02-19

**Authors:** Catherine Staton, Joao Vissoci, Enying Gong, Nicole Toomey, Rebeccah Wafula, Jihad Abdelgadir, Yi Zhao, Chen Liu, Fengdi Pei, Brittany Zick, Camille D. Ratliff, Claire Rotich, Nicole Jadue, Luciano de Andrade, Megan von Isenburg, Michael Hocker

The seventh author’s name is incorrectly spelled. The correct name is Yi Zhao.
