# Supplementary material for: Road Traffic Injury Prevention Initiatives: A Systematic Review and Metasummary of Effectiveness in Low and Middle Income Countries
Source: PLoS One. 2016 Jan 6;11(1):e0144971. doi: 10.1371/journal.pone.0144971 (PMC4703343; doi:10.1371/journal.pone.0144971)
Supplement: S4 Table — (DOCX) [file pone.0144971.s004.docx]

**PubMed Search Strategy**

| **Set** | **Strategy** | **Results** |
| --- | --- | --- |
| **#1** | "Accidents, Traffic"[Mesh] OR (("Motor Vehicles"[Mesh:NoExp] OR "Automobiles"[Mesh] OR "Motorcycles"[Mesh] OR traffic[tiab] OR vehicle[tiab] OR vehicular[tiab] OR car[tiab] OR cars[tiab] OR automobile[tiab] OR automobiles[tiab] OR motorcycle[tiab] OR motorcycles[tiab] OR taxi[tiab] OR cab[tiab] OR road[tiab] OR pedestrian[tiab] OR pedestrians[tiab]) AND (accident[tiab] OR accidents[tiab] OR injury[tiab] OR injuries[tiab] OR "Wounds and Injuries"[Mesh] OR "injuries" [Subheading])) | **57637** |
| **#2** | “Developing Countries”[Mesh] OR Africa[Mesh] OR "Central America"[Mesh] OR "Afghanistan"[Mesh] OR "Armenia"[Mesh] OR "Bangladesh"[Mesh] OR "Bhutan"[Mesh] OR "Bolivia"[Mesh] OR "Cambodia"[Mesh] OR "Comoros"[Mesh] OR "Georgia (Republic)"[Mesh] OR "Guyana"[Mesh] OR "Haiti"[Mesh] OR "India"[Mesh] OR "Indonesia"[Mesh] OR "Micronesia"[Mesh] OR Democratic People's Republic of Korea[Mesh] OR "Kosovo"[Mesh] OR "Kyrgyzstan"[Mesh] OR "Laos"[Mesh] OR "Madagascar"[Mesh] OR "Moldova"[Mesh] OR "Mongolia"[Mesh] OR "Myanmar"[Mesh] OR "Nepal"[Mesh] OR "Pakistan"[Mesh] OR "Papua theNew Guinea"[Mesh] OR "Paraguay"[Mesh] OR "Philippines"[Mesh] OR "Samoa"[Mesh] OR "Melanesia"[Mesh] OR "Sri Lanka"[Mesh] OR "Syria"[Mesh] OR "Tajikistan"[Mesh] OR "East Timor"[Mesh] OR "Ukraine"[Mesh] OR "Uzbekistan"[Mesh] OR "Vanuatu"[Mesh] OR "Vietnam"[Mesh] OR "Yemen"[Mesh] OR "Afghanistan"[tiab] OR "Armenia"[tiab] OR "Bangladesh"[tiab] OR "Bhutan"[tiab] OR "Bolivia"[tiab] OR "Cambodia"[tiab] OR "Comoros"[tiab] OR "Georgia”[tiab] OR "Guyana"[tiab] OR "Haiti"[tiab] OR "India"[tiab] OR "Indonesia"[tiab] OR "Micronesia"[tiab] OR “Korea”[tiab] OR "Kosovo"[tiab] OR "Kyrgyzstan"[tiab] OR "Laos"[tiab] OR "Madagascar"[tiab] OR Micronesia[tiab] OR "Moldova"[tiab] OR "Mongolia"[tiab] OR "Myanmar"[tiab] OR "Nepal"[tiab] OR "Pakistan"[tiab] OR "Papua New Guinea"[tiab] OR "Paraguay"[tiab] OR "Philippines"[tiab] OR "Samoa"[tiab] OR "Melanesia"[tiab] OR "Sri Lanka"[tiab] OR "Syria"[tiab] OR "Tajikistan"[tiab] OR "East Timor"[tiab] OR "Ukraine"[tiab] OR "Uzbekistan"[tiab] OR "Vanuatu"[tiab] OR "Vietnam"[tiab] OR "Yemen"[tiab] OR Africa[tiab] OR African[tiab] OR algeria[tiab] OR angola[tiab] OR benin[tiab] OR botswana[tiab] OR burkina faso[tiab] OR burundi[tiab] OR cameroon[tiab] OR cape verde[tiab] OR central african republic[tiab] OR chad[tiab] OR comoros[tiab] OR congo[tiab] OR cote d'ivoire[tiab] OR ivory coast[tiab] OR congo[tiab] OR zaire[tiab] OR Djibouti[tiab] OR egypt[tiab] OR equatorial guinea[tiab] OR ethiopia[tiab] OR eritrea[tiab] OR gabon[tiab] OR gambia[tiab] OR ghana[tiab] OR guinea[tiab] OR guinee-bissau[tiab] OR kenya[tiab] OR lesotho[tiab] OR liberia[tiab] OR libya[tiab] OR madagascar[tiab] OR malawi[tiab] OR mali[tiab] OR mauritania[tiab] OR mauritius[tiab] OR Mayotte[tiab] OR morocco[tiab] OR mozambique[tiab] OR namibia[tiab] OR niger[tiab] OR nigeria[tiab] OR reunion[tiab] OR rwanda[tiab] OR sahara[tiab] OR saint Helena[tiab] OR sao tome[tiab] OR senegal[tiab] OR seychelles[tiab] OR sierra leone[tiab] OR somalia[tiab] OR south africa[tiab] OR sudan[tiab] OR swaziland[tiab] OR togo[tiab] OR tanzania[tiab] OR tunisia[tiab] OR uganda[tiab] OR zambia[tiab] OR zimbabwe[tiab] OR georgia[tiab] OR "solomon islands"[tiab] OR "west bank"[tiab] OR "gaza"[tiab] OR kiribati[tiab] OR "El Salvador"[tiab] OR "cabo verde"[tiab] OR guatemala[tiab] OR honduras[tiab] OR nicaragua[tiab] OR korea[tiab] OR "kyrgyz"[tiab] OR laos[tiab] OR "low resource"[tiab] OR "under-resourced"[tiab] OR "resource poor"[tiab] OR "under-developed"[tiab] OR "underdeveloped"[tiab] OR "developing country"[tiab] OR "developing countries"[tiab] OR "developing world"[tiab] OR “third world”[tiab] OR lmic[tiab] OR (low[tiab] AND middle[tiab] AND income[tiab]) | **715840** |
| **#3** | **#1 AND #2** | **2732** |
