# Supplementary material for: Road Traffic Injury Prevention Initiatives: A Systematic Review and Metasummary of Effectiveness in Low and Middle Income Countries
Source: PLoS One. 2016 Jan 6;11(1):e0144971. doi: 10.1371/journal.pone.0144971 (PMC4703343; doi:10.1371/journal.pone.0144971)
Supplement: S5 Table — (DOCX) [file pone.0144971.s005.docx]

**Embase Search Strategy**

| **Set** | **Strategy** | **Results** |
| --- | --- | --- |
| **#1** | 'traffic accident'/exp OR (('motor vehicle'/exp OR 'pedestrian'/exp OR 'traffic'/exp OR traffic:ab,ti OR vehicle:ab,ti OR vehicular:ab,ti OR car:ab,ti OR cars:ab,ti OR automobile:ab,ti OR automobiles:ab,ti OR motorcycle:ab,ti OR motorcycles:ab,ti OR taxi:ab,ti OR cab:ab,ti OR road:ab,ti OR pedestrian:ab,ti OR pedestrians:ab,ti) AND ('injury'/exp OR accident:ab,ti OR accidents:ab,ti OR injury:ab,ti OR injuries:ab,ti)) | **83810** |
| **#2** | 'developing country'/exp OR 'Africa'/exp OR 'Central America'/exp OR 'Afghanistan'/exp OR 'Armenia'/exp OR 'Bangladesh'/exp OR 'Bhutan'/exp OR 'Bolivia'/exp OR 'Cambodia'/exp OR 'Comoros'/exp OR 'Georgia (republic)'/exp OR 'Guyana'/exp OR 'Haiti'/exp OR 'India'/exp OR 'Indonesia'/exp OR 'Federated States of Micronesia'/exp OR 'North Korea'/exp OR 'Kosovo'/exp OR 'Kyrgyzstan'/exp OR 'Laos'/exp OR 'Madagascar'/exp OR 'Moldova'/exp OR 'Mongolia'/exp OR 'Myanmar'/exp OR 'Nepal'/exp OR 'Pakistan'/exp OR 'Papua New Guinea'/exp OR 'Paraguay'/exp OR 'Philippines'/exp OR 'Samoan IslORs'/exp OR 'Melanesia'/exp OR 'Sri Lanka'/exp OR 'Syrian Arab Republic'/exp OR 'Tajikistan'/exp OR 'Timor-Leste'/exp OR 'Ukraine'/exp OR 'Uzbekistan'/exp OR 'Vanuatu'/exp OR 'Viet Nam'/exp OR 'Yemen'/exp OR ‘Afghanistan’:ab,ti OR ‘Armenia’:ab,ti OR ‘Bangladesh’:ab,ti OR ‘Bhutan’:ab,ti OR ‘Bolivia’:ab,ti OR ‘Cambodia’:ab,ti OR ‘Comoros’:ab,ti OR ‘Georgia’:ab,ti OR ‘Guyana’:ab,ti OR ‘Haiti’:ab,ti OR ‘India’:ab,ti OR ‘Indonesia’:ab,ti OR ‘Micronesia’:ab,ti OR ‘Korea’:ab,ti OR ‘Kosovo’:ab,ti OR ‘Kyrgyzstan’:ab,ti OR ‘Laos’:ab,ti OR ‘Madagascar’:ab,ti OR Micronesia:ab,ti OR ‘Moldova’:ab,ti OR ‘Mongolia’:ab,ti OR ‘Myanmar’:ab,ti OR ‘Nepal’:ab,ti OR ‘Pakistan’:ab,ti OR ‘Papua New Guinea’:ab,ti OR ‘Paraguay’:ab,ti OR ‘Philippines’:ab,ti OR ‘Samoa’:ab,ti OR ‘Melanesia’:ab,ti OR ‘Sri Lanka’:ab,ti OR ‘Syria’:ab,ti OR ‘Tajikistan’:ab,ti OR ‘East Timor’:ab,ti OR ‘Ukraine’:ab,ti OR ‘Uzbekistan’:ab,ti OR ‘Vanuatu’:ab,ti OR ‘Vietnam’:ab,ti OR ‘Yemen’:ab,ti OR Africa:ab,ti OR African:ab,ti OR algeria:ab,ti OR angola:ab,ti OR benin:ab,ti OR botswana:ab,ti OR ‘burkina faso’:ab,ti OR burundi:ab,ti OR cameroon:ab,ti OR ‘cape verde’:ab,ti OR ‘central african republic’:ab,ti OR chad:ab,ti OR comoros:ab,ti OR congo:ab,ti OR ‘cote d ivoire’:ab,ti OR ‘ivory coast’:ab,ti OR congo:ab,ti OR zaire:ab,ti OR Djibouti:ab,ti OR egypt:ab,ti OR ‘equatorial guinea’:ab,ti OR ethiopia:ab,ti OR eritrea:ab,ti OR gabon:ab,ti OR gambia:ab,ti OR ghana:ab,ti OR guinea:ab,ti OR ‘guinee bissau’:ab,ti OR kenya:ab,ti OR lesotho:ab,ti OR liberia:ab,ti OR libya:ab,ti OR madagascar:ab,ti OR malawi:ab,ti OR mali:ab,ti OR mauritania:ab,ti OR mauritius:ab,ti OR Mayotte:ab,ti OR morocco:ab,ti OR mozambique:ab,ti OR namibia:ab,ti OR niger:ab,ti OR nigeria:ab,ti OR reunion:ab,ti OR rwanda:ab,ti OR sahara:ab,ti OR ‘saint Helena’:ab,ti OR ‘sao tome’:ab,ti OR senegal:ab,ti OR seychelles:ab,ti OR ‘sierra leone’:ab,ti OR somalia:ab,ti OR ‘south africa’:ab,ti OR sudan:ab,ti OR swaziland:ab,ti OR togo:ab,ti OR tanzania:ab,ti OR tunisia:ab,ti OR uganda:ab,ti OR zambia:ab,ti OR zimbabwe:ab,ti OR georgia:ab,ti OR ‘solomon islands’:ab,ti OR ‘west bank’:ab,ti OR ‘gaza’:ab,ti OR kiribati:ab,ti OR ‘El Salvador’:ab,ti OR ‘cabo verde’:ab,ti OR guatemala:ab,ti OR honduras:ab,ti OR nicaragua:ab,ti OR korea:ab,ti OR kyrgyz:ab,ti OR laos:ab,ti OR ‘low resource’:ab,ti OR ‘under resourced’:ab,ti OR ‘resource poor’:ab,ti OR ‘under developed’:ab,ti OR ‘underdeveloped’:ab,ti OR ‘developing country’:ab,ti OR ‘developing countries’:ab,ti OR ‘developing world’:ab,ti OR ‘third world’:ab,ti OR lmic:ab,ti OR (low:ab,ti AND middle:ab,ti AND income:ab,ti) | **870432** |
| **#3** | **#1 AND #2** | **3516** |
