# Supplementary material for: Road Traffic Injury Prevention Initiatives: A Systematic Review and Metasummary of Effectiveness in Low and Middle Income Countries
Source: PLoS One. 2016 Jan 6;11(1):e0144971. doi: 10.1371/journal.pone.0144971 (PMC4703343; doi:10.1371/journal.pone.0144971)
Supplement: S6 Table — (DOCX) [file pone.0144971.s006.docx]

**Scopus Search Strategy**

| **Set #** |  | **Results** |
| --- | --- | --- |
| **1** | TITLE-ABS-KEY( (("Motor Vehicles" OR Automobiles OR Motorcycles OR traffic OR vehicle OR vehicular OR car OR cars OR automobile OR motorcycle OR taxi OR cab OR road OR pedestrian OR pedestrians) AND (accident OR accidents OR injury OR injuries))) | **114986** |
| **2** | TITLE-ABS-KEY(“developing country” OR “developing countries” OR "Afghanistan" OR "Armenia" OR "Bangladesh" OR "Bhutan" OR "Bolivia" OR "Cambodia" OR "Comoros" OR "Georgia” OR "Guyana" OR "Haiti" OR "India" OR "Indonesia" OR "Micronesia" OR “Korea” OR "Kosovo" OR "Kyrgyzstan" OR "Laos" OR "Madagascar" OR “Micronesia” OR "Moldova" OR "Mongolia" OR "Myanmar" OR "Nepal" OR "Pakistan" OR "Papua New Guinea" OR "Paraguay" OR "Philippines" OR "Samoa" OR "Melanesia" OR "Sri Lanka" OR "Syria" OR "Tajikistan" OR "East Timor" OR "Ukraine" OR "Uzbekistan" OR "Vanuatu" OR "Vietnam" OR "Yemen" OR “Africa” OR “African” OR algeria OR angola OR benin OR botswana OR “burkina faso” OR burundi OR cameroon OR “cape verde” OR “central african republic” OR chad OR comoros OR congo OR “cote d'ivoire” OR “ivory coast” OR congo OR zaire OR Djibouti OR egypt OR “equatorial guinea” OR ethiopia OR eritrea OR gabon OR gambia OR ghana OR guinea OR “guinee-bissau” OR kenya OR lesotho OR liberia OR libya OR madagascar OR malawi OR mali OR mauritania OR mauritius OR Mayotte OR morocco OR mozambique OR namibia OR niger OR nigeria OR reunion OR rwanda OR sahara OR “saint Helena” OR “sao tome” OR senegal OR seychelles OR “sierra leone” OR somalia OR “south Africa” OR sudan OR swaziland OR togo OR tanzania OR tunisia OR uganda OR zambia OR zimbabwe OR georgia OR "solomon islands" OR "west bank" OR "gaza" OR kiribati OR "El Salvador" OR "cabo verde" OR guatemala OR honduras OR nicaragua OR korea OR "kyrgyz" OR laos OR "low resource" OR "under-resourced" OR "resource poor" OR "under-developed" OR "underdeveloped" OR "developing world" OR “third world” OR lmic OR (low AND middle AND income)) | **1737966** |
| **3** | #1 AND #2 | **4729** |
| **4** | #4, Limits: 2005 - present | **(determine duplicates from total 3571)** |
